# The Impact of Data Vulnerability in Online Health Communities: An Institutional Assurance Perspective

**DOI:** 10.3389/fpsyg.2022.908309

**Published:** 2022-06-29

**Authors:** Wei Gao, Huiling Wang, Ning Jiang

**Affiliations:** ^1^College of Economics and Management, Southwest University, Chongqing, China; ^2^Antai College of Economics and Management, Shanghai Jiao Tong University, Shanghai, China

**Keywords:** data vulnerability, psychological comfort, institutional assurance approach, online health communities, continuance intention

## Abstract

In the last few years, online health communities (OHCs) have experienced rapid development due to advances in technology and the COVID-19 pandemic. However, the sensitive nature of medical information has also raised concerns from users about their privacy and reduced their intention to use OHCs. Considering the critical role of data privacy, this study explored the effect of data vulnerability on OHC users. Using online survey data collected from 438 OHC users in China, we found that data vulnerability significantly reduced psychological comfort, while the latter enhanced continuance intention. We also found that psychological comfort negatively mediated the impact of data vulnerability on continuance intention. Institutional assurance approaches, namely privacy policy, privacy protection technology, industry self-regulation, and government legislation, were also found to mitigate the negative impact of data vulnerability on psychological comfort. This study not only contributes to the data privacy, psychological comfort, and institutional assurance literature but also offers suggestions for OHC stakeholders.

## Introduction

With the rapid development of digital information technologies, such as artificial intelligence, 5G, and big data analytics, medical and health information services on the Internet are becoming increasingly popular. In China, the number of online health or medical consultations rose by 49% from 2012 to 2016 and is likely to experience accelerated growth in the coming years, potentially surpassing 1.5 billion annually by 2025 (McKinsey, 2020). In addition, there is a growing number of people who participate in online health communities (OHCs) to search for health information and self-care advice (Zhang et al., 2018). An OHC is a “virtual social network where individuals can share health experiences, post health questions, seek, and/or provide support” (Chen et al., 2019, p. 195). Compared with traditional offline medical and health information services, OHCs not only enable individuals to acquire more abundant and diverse health information without the restrictions of time and space (Zhang et al., 2018; Virlée et al., 2020), but also reduce the perceived severity of their disease and enhance their quality of life (Tseng et al., 2022). Thus, OHCs have become useful channels for people seeking health information over the last dozen years (Zhang et al., 2018; Liu et al., 2020; Zhou, 2020; James et al., 2022). Especially because of the COVID-19 pandemic, people are increasingly acknowledging the benefits of OHCs and becoming users (Zhang et al., 2021; Shao et al., 2022).

Although OHCs have widely recognized advantages, their users face a higher risk of personal data vulnerability than in other online communities because they usually disclose sensitive information, such as their health information and medical records, to seek medical help. Data vulnerability is defined as individuals’ feelings of susceptibility to harm due to unwanted uses of their personal data (Martin et al., 2017). In this digital age, people have high awareness of information security (Mamonov and Benbunan-Fich, 2018; Mohr and Walter, 2019; Mombeuil and Uhde, 2021) and are increasingly emphasizing data privacy (Wang et al., 2019; Esmaeilzadeh, 2020b; Liyanaarachchi, 2021; Martínez-Navalón et al., 2021). A likely reason is that information leakage or data breach events have occurred frequently in recent years (Goode et al., 2017). In particular, medical data leaks, which are highly sensitive, can result in substantial psychological harm and deter individuals from using online health services (Hua et al., 2019; Maxwell and Grupac, 2021; Turner and Pera, 2021). When people use OHC services, they are concerned that their sensitive personal information may be leaked or misused without authorization (Zhang et al., 2018). This concern (i.e., data vulnerability) is likely to affect their intention to use in OHCs. Thus, how to manage user data safely and effectively and thereby decrease their data vulnerability is a critical issue for OHCs to solve.

Although researchers have illustrated the importance of data privacy protection or information security for online users (Wang et al., 2019; Yang et al., 2020; Tsai and Tiwasing, 2021), no study has examined the influence of data vulnerability on OHC users. Moreover, to the best of our knowledge, no study has explored how institutional assurance may reduce data vulnerability. Therefore, to fill these research gaps, this study investigated the following two interrelated research questions. First, how does OHC users’ data vulnerability affect their psychological comfort and continuance intention? Second, are the institutional assurance approaches of privacy policy, privacy protection technology, industry self-regulation, and government legislation effective in mitigating the negative impact of data vulnerability on OHC users?

More precisely, this research contributes to the literature in the following three aspects. First, although some studies have examined the effect of data vulnerability and promoted scrutiny of corporate data management practices over that user privacy concerns as a solution (Liyanaarachchi et al., 2021), data vulnerability in the context of OHCs has not been explored. By introducing the concept of data vulnerability in the study of OHCs, this study extends the contexts in which data vulnerability is researched.

Second, this research examined the mediating role of psychological comfort in the relationship between data vulnerability and continuance intention. Therefore, it provides a way to understand the mechanism by which data vulnerability operates from the perspective of psychological comfort. It enriches knowledge on data vulnerability and extends the comfort literature to include the OHC research context.

Third, this study identified and explored four data vulnerability suppressors from the perspective of institutional assurance. Although Martin et al. (2017) suggested that transparency and perceived control could effectively mitigate the negative impact of data vulnerability, no research has considered the impact of institutional mitigation strategies. As we examined how privacy policy, privacy protection technology, industry self-regulation, and government legislation could moderate the effect of data vulnerability on psychological comfort, this study not only facilitates a deeper understanding of data vulnerability but also a more holistic approach to governing relevant issues.

## Research Background

### Data Vulnerability

Vulnerability refers to an individual’s susceptibility to harm or injury (Smith and Cooper-Martin, 1997) and reflects an object’s inherent weakness (Lee, 2020). Research has found that consumer vulnerability (Baker et al., 2005; Kim, 2019; Glavas et al., 2020), financial vulnerability (O’Connor et al., 2019), and technology vulnerability (Lee, 2020) can have a negative impact on individuals. However, limited research has focused on the effects of data vulnerability, which refers to individuals’ feelings of susceptibility to harm due to an organization’s possession of their personal data (Martin et al., 2017). When an organization collects, stores, and uses the personal data of users, the possibility of data misuse enhances their vulnerability. That is, users perceive greater data vulnerability when they lose control of their personal data (Warner and Wang, 2019; Liyanaarachchi et al., 2021).

Individuals may be more vulnerable when they use an OHC rather than traditional offline medical services, because their online personal data can be easily disclosed, misused, and even sold to third parties by the OHC (Warner and Wang, 2019). In addition, Martin et al. (2017) found that data vulnerability could increase individuals’ feelings of violation, decrease their trust in the organization holding their data, and worsen firm performance. Therefore, it is important for OHCs to explore how data vulnerability affects their users and how they may reduce the negative impact of data vulnerability effectively.

### Psychological Comfort

Psychological comfort refers to individuals’ emotional feelings of comfort and security toward disclosing personal data to others (Yang et al., 2020). In this study, it refers to OHC users’ emotional feelings of comfort in revealing their personal data when using OHCs (Sutanto et al., 2013). High levels of psychological comfort indicate that individuals are not concerned about the security of their personal data (Robinson, 2018). Research has found that psychological comfort can induce positive outcomes, such as users’ higher intention to disclose personal information (Yang et al., 2020), save advertisements intention (Sutanto et al., 2013), and repeat purchase intentions (Akhtar et al., 2019). Thus, psychological comfort is an important predictor of individuals’ behavioral intentions, and its improvement is critical for the success of OHCs.

### Institutional Assurance Approaches

Institutional assurance is regarded as a salient environmental factor that affects individuals’ decisions to disclose their personal information (Xu et al., 2011). It refers to interventions made by powerful agents to assure individuals that considerable effort has been invested in protecting their data privacy and that the organizations holding their data have fair and reasonable data practices (McKnight et al., 2002; Xu et al., 2011, 2012). These powerful agents include service providers, industry regulators, and government (Gong et al., 2019; Yang et al., 2020). Specifically, previous pieces of literature propose four types of institutional assurance approaches, such as privacy policy, privacy protection technology, industry self-regulation, and government legislation (Zhao et al., 2012; Gong et al., 2019; Wang et al., 2019), all of which were examined in this research.

Privacy policy and privacy protection technology are institutional assurance approaches provided by service providers (Culnan and Bies, 2003; Wang et al., 2019). A privacy policy is a statement that details the service provider’s promise to protect and manage individuals’ personal data (Karwatzki et al., 2017). The privacy policy informs users of what data will be collected, and how the data will be used. Privacy protection technology refers to technological measures that protect the security of individuals’ personal data (Wang et al., 2019). Industry self-regulation is established by independent industry groups or certifying agencies (Xu et al., 2011) and consists of the rules and enforcement procedures developed by industries to ensure companies’ fair data practices (Culnan and Bies, 2003). Government legislation refers to the measures made by a government’s judicial and legislative branches to protect individuals’ personal data (Gong et al., 2019). All of these institutional assurance approaches are critical for solving data privacy issues, but little research has integrated and examined the four types simultaneously. Therefore, this study investigated the moderating effects of privacy policy, privacy protection technology, industry self-regulation, and government legislation in the context of OHCs.

## Conceptual Model and Hypotheses

**FIGURE 1:**
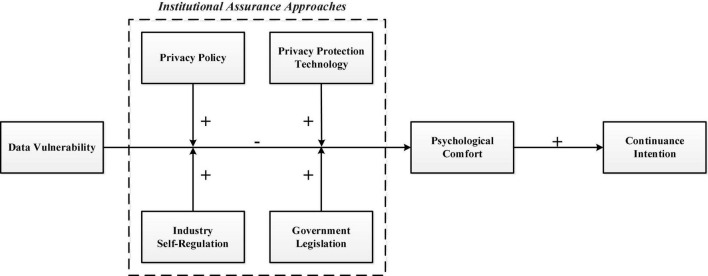
Conceptual model.

The conceptual model of this study is shown in Figure 1. We first explored the effect of data vulnerability on psychological comfort and the effect of psychological comfort on continuance intention. We then examined the mediating role of psychological comfort in the relationship between data vulnerability and continuance intention. Finally, we explored the moderating role of four institutional assurance approaches (i.e., privacy policy, privacy protection technology, industry self-regulation, and government legislation) in the relationship between data vulnerability and psychological comfort.

### The Impact of Data Vulnerability on Psychological Comfort and Continuance Intention

Data vulnerability involves feelings of violation (Martin et al., 2017), which cause negative responses (Liyanaarachchi et al., 2021). In the OHC environment, data vulnerability indicates that the users feel insecure, vulnerable, and exposed since the OHCs have their personal health data, including disease-related information and medical records. This perception of vulnerability makes OHC users feel susceptible (Martin et al., 2017), because the leaks of these data can result in reputation damage, mental stress, embarrassment, blackmail, disease discrimination, and identity theft (Slepchuk et al., 2022; Sun et al., 2022; Wu et al., 2022). Hence, the OHC users may not feel comfortable providing their sensitive data to the OHCs. In addition, the OHCs that have users’ medical data can also suffer a security lapse, such as data breach, which put the users in a risky situation (Wu et al., 2022). Consequently, a high level of data vulnerability indicates that OHC users are concerned their personal data will be associated with great unauthorized access and illegal use (Dinev et al., 2013; Sun et al., 2022). These concerns for their personal data security will make OHC users feel uncomfortable and anxiety (Sutanto et al., 2013; Robinson, 2018), and their psychological contracts with OHCs will be breached (Liu et al., 2021). We accordingly propose the following hypothesis:

**H1: Data vulnerability is negatively related to psychological comfort.**

Psychological comfort is a state of mind where individuals feel calm and at ease (Spake et al., 2003) and reflects a feeling of worry-free in an environment (Radia et al., 2022). In this study, psychological comfort demonstrates the comfortable feeling of disclosing personal data when individuals use the service of OHC (Yang et al., 2020). When OHC users feel reliable and comfortable providing personal data to the OHCs, the relationship quality between users and service providers of OHCs will be enhanced (Mahmood and Tayib, 2021; Radia et al., 2022). Furthermore, the improved psychological comfort indicates reduced psychological tension, which suggests the OHC users are worry-free concerning service encounters with the service provider of OHCs. So, psychological promotes OHC users to go beyond the available cognitive pieces of evidence to feel at ease and assured toward continued using OHC (Dinev et al., 2015). In addition, according to the comfort pieces of literature, comfortable psychological state is an important emotional motivator that can induce positive outcomes, such as repeat purchases (Liang et al., 2018), positive word of mouth (Akhtar et al., 2019), disclosing personal information (Yang et al., 2020), and purchase intention (Otterbring et al., 2021). In the context of an OHC, high degree of psychological comfort suggests that users perceive the provision of personal data to an OHC as being emotional comfortable, which then enhances the users’ continuance intention to adopt the OHC. Therefore, we propose the following hypothesis:

**H2: Psychological comfort is positively related to continuance intention.**

Combining the logic of H1 and H2, we conjecture that psychological comfort plays an important role in the relationship between data vulnerability and continuance intention. When OHC users perceive a high level of data vulnerability, they are likely to feel uncomfortable disclosing their health-related information. Given their lack of confidence in the OHCs’ ability and willingness to protect their data security, they are likely to be reluctant to continue using the service of OHCs. Besides, psychological comfort has been identified as important mediating construct (e.g., Yang et al., 2020; Radia et al., 2022). Therefore, we propose the following hypothesis:

**H3: Psychological comfort negatively mediates the impact of data vulnerability on continuance intention.**

### The Moderating Effect of Institutional Assurance Approaches

When OHC users perceive a privacy policy to be highly effective, they believe that it provides reliable information about how the OHC collects and uses their personal data (Xu et al., 2011; Yang et al., 2020). The privacy policy therefore offers users the promise to be able to securely disclose their personal data and use OHC services (Gong et al., 2019; Zeng et al., 2022). Under these circumstances, users perceive a high level of information control, which can mitigate their feelings of violation and promote trust-related behaviors (Esmaeilzadeh, 2020a; Mutimukwe et al., 2020; Guo et al., 2022). Moreover, the principles of privacy policy should demonstrate fair information practices (Culnan, 2019), which can effectively decrease privacy concerns (Nikkhah and Sabherwal, 2021) and increase the willingness to disclose personal data (Liu et al., 2022). Therefore, the perception of insecure and vulnerable associated with data vulnerability (Liyanaarachchi et al., 2021) may be reduced through the establishment of an effective privacy policy. We accordingly propose the following hypothesis:

**H4: Privacy policy positively moderates the relationship between data vulnerability and psychological comfort.**

Privacy protection technology offered by service providers allows users to make personalized technological choices to guarantee the safety of their personal data online (Duan and Wang, 2015). OHCs that adopt privacy protection technology can decrease the risk of unauthorized or improper personal data access, thus reducing their users’ data vulnerability (Van der Schyff et al., 2020). Some privacy protection technologies can also maintain the anonymity of users’ data and allow them to adjust the level and scope of personal data available to other parties (Wang and Li, 2021). In addition, advanced mainstream cryptography and blockchain technology can facilitate the safe storage and transfer of health data (Abdellatif et al., 2021). Therefore, users may perceive high levels of control over their personal data in OHCs with highly effective privacy protection technology (Wang et al., 2019). Martin et al. (2017) suggested that the perception of information control could reduce the negative impact of data vulnerability on cognitive trust. Hence, effective privacy protection technology could weaken the negative effect of data vulnerability on psychological comfort through offering OHC users perceived control over their personal data. Furthermore, various privacy protection technologies empower individuals to deal with firms’ data collection practices and provide them with more choices to protect their data privacy (Wang et al., 2019). Therefore, we propose the following hypothesis:

**H5: Privacy protection technology positively moderates the relationship between data vulnerability and psychological comfort.**

Industry self-regulation refers to the enforcement procedures and rules developed by industry regulators to enhance the trustworthiness of companies in that industry (Xu et al., 2012). The independent industry regulator is responsible for protecting the data privacy of users in relation to personal data collection and usage (Gong et al., 2019). Effective industry self-regulation for OHCs entails fair monitoring and governance of their data practices and enables users to form positive expectations of these practices (Hemphill and Banerjee, 2021). Thus, when industry self-regulation is high, OHC users perceive a low risk of data misuse and high levels of information control (Xu et al., 2011). The negative impact of data vulnerability on psychological comfort may thus be weakened. What’s more, industry self-regulation signals to users that the service providers of OHCs will not behave opportunistically with their personal data (Nikkhah and Sabherwal, 2021). Even if their data security is compromised, the industry self-regulation programs will provide recourse (Xu et al., 2009). Therefore, we propose the following hypothesis:

**H6: Industry self-regulation positively moderates the relationship between data vulnerability and psychological comfort.**

In this study, government legislation refers to the measures enacted by the legislative and judicial branches of a government to protect the safety of individuals’ personal data (Xu et al., 2012; Gong et al., 2019). It indicates that the government, as an independent third party, will protect users’ sensitive personal data through effective regulation of organizations’ data practices (Gong et al., 2019; Hong et al., 2021). Thus, when there is highly effective government legislation, OHC users may perceive a lower risk of data misuse. In addition, the strict government legislations indicate that the OHCs will pay a high price when behaving opportunistically with users’ personal data (Hong et al., 2021). Therefore, OHC users could have high-level control of their own data, and then, the negative impact of data vulnerability on psychological comfort may be mitigated by effective government legislation. Moreover, research has found that effective government laws and regulations are important forms of assurance that can reduce individuals’ data privacy concerns by offering them high-level control of their personal data (Lee, 2020; Hong et al., 2021). Hence, we propose the following hypothesis:

**H7: Government legislation positively moderates the relationship between data vulnerability and psychological comfort.**

## Research Methods

### Sample and Data Collection

In this study, we focused on general-purpose OHCs in China, such as Dingxiangyuan^1^, Haodaifu^2^, and Xunyiwenyao^3^. To test our conceptual model and hypotheses, we conducted an online survey to collect data through the professional third-party platform Sojump, which provides access to large samples in China. Before sending out the questionnaire, we invited experts to check its terminology, logical consistency, and clarity. We then modified our questionnaire according to their advice and formulated the final version. The questionnaire consisted of four parts. The first section introduced the purpose of this research and guaranteed the anonymity and absolute confidentiality of the participants’ information. The second section collected the participants’ demographic information, namely their gender, age, education, and monthly income. In the third section, we asked the participants whether they had experience using OHCs. Only the participants who had registered with and used OHCs could continue filling in the questionnaire. These participants were asked to choose one OHC that they had used and answer the subsequent questions based on their selected OHC. The fourth section presented the core constructs of our study. Our final sample consisted of 438 valid responses obtained in 2 weeks. Of the participants, 51.4% were male, 50.2% were between 31 and 40 years old, and 81.5% had a bachelor’s degree or above (see Table 1). To check for non-response bias, we compared the first 25% and last 25% of the participants. Our results indicated that non-response bias was not a major issue in this study.

**TABLE 1:** Demographic information.

	Category	Number	Frequency
Gender	Male	225	51.4
	Female	213	48.6
Age	≤30	152	34.7
	31–40	220	50.2
	≥41	66	15.1
Education	Junior college and below	81	18.5
	Bachelor	297	67.8
	Master	54	12.3
	Doctoral	6	1.4
Monthly income (Yuan)	≤3000	79	18.1
	3001–6000	173	39.5
	6001–9000	96	21.9
	9001–12000	82	18.7
	>12000	8	1.8

## Measurements

All of the measures in this research were adapted from previous studies (see Supplementary Appendix). The items for data vulnerability were adapted from Martin et al. (2017); the three items for psychological comfort were adapted from Yang et al. (2020); the three items for privacy policy were adapted from Xu et al. (2011); the items for privacy protection technology were adapted from Wang et al. (2019); the three items for industry self-regulation were adapted from Xu et al. (2011); the three items for government legislation were adapted from Gong et al. (2019); and the four items for continuance intention were adapted from Kim et al. (2019). All of the items were scored on a seven-point Likert scale. As seen in Table 2, the means of the core constructs ranged from 4.283 to 5.198, and the standard deviations ranged from 1.137 to 1.766. We controlled for gender (mean = 0.510, SD = 0.500), age (mean = 33.610, SD = 5.826), education (mean = 1.970, SD = 0.602), and monthly income (mean = 2.468, SD = 1.047) as the control variables.

**TABLE 2:** Correlations.

Variables	1	2	3	4	5	6	7	8	9	10	11
1. Data vulnerability	**0.858**										
2. Psychological comfort	−0.210**	**0.875**									
3. Privacy policy	0.090	0.084	**0.863**								
4. Privacy protection technology	−0.041	0.088	0.054	**0.851**							
5. Industry Self-Regulation	0.007	0.113*	0.131**	0.075	**0.855**						
6. Government legislation	0.065	0.136**	0.037	0.036	0.067	**0.847**					
7. Continuance Intention	−0.046	0.242**	0.046	0.132**	0.162**	0.061	**0.777**				
8. Gender	−0.074	0.067	−0.069	−0.072	−0.092	−0.088	−0.054	**NA**			
9. Age	0.011	0.111*	0.089	0.093	0.043	0.063	0.144**	−0.036	**NA**		
10. Education	−0.042	−0.144**	0.006	−0.138**	−0.010	−0.020	0.026	−0.048	−0.073	**NA**	
11. Monthly income	−0.010	0.017	−0.006	−0.049	−0.003	−0.105*	−0.024	0.051	−0.127**	0.167**	**NA**
**Mean**	4.939	4.283	4.940	4.873	4.941	4.885	5.198	0.510	33.610	1.970	2.468
**SD**	1.431	1.766	1.632	1.596	1.586	1.556	1.137	0.500	5.826	0.602	1.047

*p < 0.05, **p < 0.01. The diagonal numbers in bold are the square roots of AVEs. NA represents not applicable.

### Measurement Assessment

We conducted a confirmatory factor analysis (CFA) to test the reliability and validity of our measures. The CFA results indicated that our data had a good fit with the conceptual model (χ^2^ = 416.992, df = 231, RMSEA = 0.043, CFI = 0.972, TLI = 0.967, SRMR = 0.030). As presented in Table 3, all of the Cronbach’s α and composite reliability (CR) values were also greater than 0.7, suggesting that all of the variables had good reliability (Fornell and Larcker, 1981). The average variance extracted (AVE) scores were greater than 0.5, indicating that the constructs had adequate convergent validity (Fornell and Larcker, 1981). As shown in Table 2, the square roots of the AVE for all of the variables were greater than the correlation coefficients, indicating good discriminant validity (Fornell and Larcker, 1981). In addition, following the method recommended by Lindell and Whitney (2001) to examine common method variance (CMV), we adjusted the correlations according to the lowest positive correlation (*r* = 0.006; see Table 2) and compared the results with the unadjusted correlation matrix. The original significant correlations maintained their significance, indicating that CMV was not a serious issue in this research.

**TABLE 3:** Reliability and validity.

Constructs	Item	Loading	Cronbach’s α	CR	AVE
Data vulnerability	DVU1	0.848	0.933	0.933	0.737
	DVU2	0.822			
	DVU3	0.851			
	DVU4	0.887			
	DVU5	0.883			
Psychological comfort	PCO1	0.854	0.907	0.908	0.766
	PCO2	0.908			
	PCO3	0.863			
Privacy policy	PPO1	0.817	0.896	0.897	0.744
	PPO2	0.926			
	PPO3	0.840			
Privacy protection technology	PPT1	0.869	0.885	0.888	0.725
	PPT2	0.892			
	PPT3	0.790			
Industry self-regulation	ISR1	0.861	0.890	0.891	0.731
	ISR2	0.885			
	ISR3	0.818			
Government legislation	GLE1	0.825	0.883	0.884	0.718
	GLE2	0.866			
	GLE3	0.851			
Continuance intention	CIN1	0.727	0.856	0.858	0.603
	CIN2	0.819			
	CIN3	0.838			
	CIN4	0.714			

## Analysis Results

Mplus 7 was used to analyze the data. The multiple regression results are presented in Table 4. The result of Model 1 showed that the coefficient between data vulnerability and psychological comfort was significantly negative (β = −0.280, *p* < 0.001), indicating that data vulnerability had a negative effect on the participants’ psychological comfort. H1 was thus supported. In Model 3, the coefficient between psychological comfort and continuance intention was significant and positive (β = 0.143, *p* < 0.001), suggesting that psychological comfort positively affected continuance intention. H2 was thus supported.

**TABLE 4:** Regression results.

Variables	Psychological comfort		Continuance intention
	Model 1	Model 2	Model 3
Constant	3.804***	3.465***	3.676***
Gender	0.259	0.098	–0.092
Age	0.027*	0.040**	0.021*
Education	−0.425**	−0.375**	0.156*
Income	0.109	0.058	–0.022
PPO	0.090	0.120*	–0.006
PPT	0.046	0.025	0.072
ISR	0.103	0.101	0.088*
GLE	0.164**	0.175**	0.006
DVU	−0.280***	−0.274***	0.003
DVU × PPO		0.086***	
DVU × PPT		0.085*	
DVU × ISR		0.077*	
DVU × GLE		0.078**	
PCO			0.143***
*R* ^2^	0.125	0.193	0.107
***Δ** R^2^*		0.068***	
*F-statistics*	6.808***	7.802***	5.131***

*p < 0.05, **p < 0.01, ***p < 0.001.

To test the mediating effect of psychological comfort, we adopted the bootstrap method recommended by Zhao et al. (2010). First, to calculate the mediating effect (−0.040 = −0.280 × 0.143), we multiplied the coefficient between data vulnerability and psychological comfort (−0.280) by the coefficient between psychological comfort and continuance intention (0.143). Then, we performed a bootstrap analysis with 5000 resamples to test the significance of the mediating effect of psychological comfort. The result showed that the 95% bias-corrected confidence interval did not contain zero (−0.069, −0.020), suggesting that data vulnerability had a negative mediating effect on the participants’ continuance intention through psychological comfort. H3 was thus supported.

**FIGURE 2:**
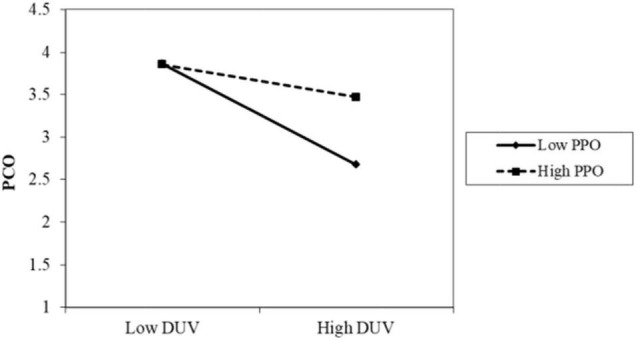
Moderating effect of PPO. PPO, privacy policy; DUV, data vulnerability; PCO, psychological comfort.

**FIGURE 3:**
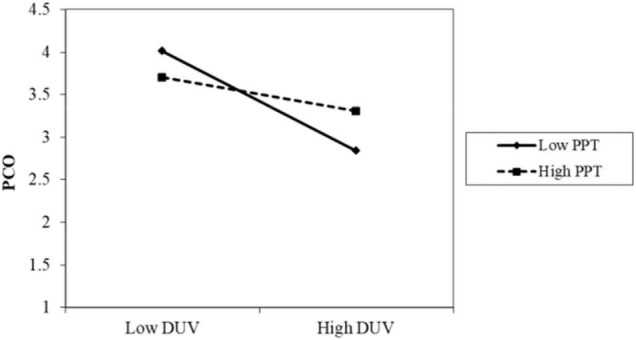
Moderating effect of PPT. PPT, privacy protection technology; DUV, data vulnerability; PCO, psychological comfort.

**FIGURE 4:**
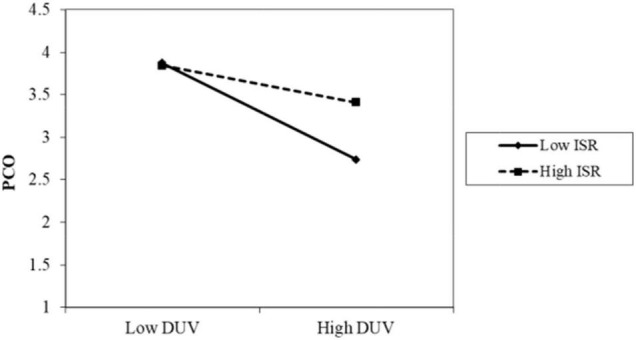
Moderating effect of ISR. ISR, industry self-regulation; DUV, data vulnerability; PCO, psychological comfort.

**FIGURE 5:**
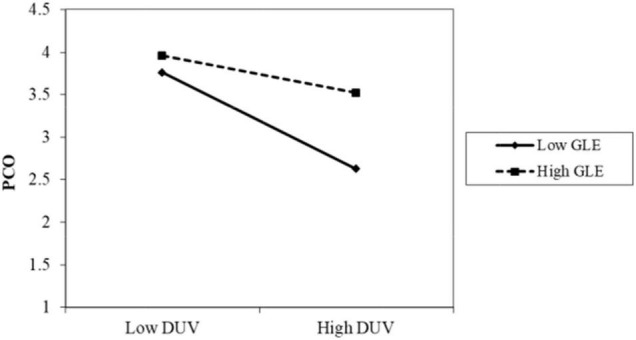
Moderating effect of GLE. GLE, government legislation; DUV, data vulnerability; PCO, psychological comfort.

The results of Model 2 are as follows. The interaction term between privacy policy and data vulnerability was significantly positive (β = 0.086, *p* < 0.001). As shown in Figure 2, when the participants perceived a high level of privacy policy implementation, the negative impact of data vulnerability on psychological comfort was reduced. Thus, H4 was supported. The interaction term between privacy protection technology and data vulnerability was also positive and significant (β = 0.085, *p* < 0.05). As shown in Figure 3, when the participants perceived a high level of privacy protection technology implementation, the negative impact of data vulnerability on psychological comfort was reduced. Therefore, H5 was supported. The interaction term between industry self-regulation and data vulnerability was similarly significantly positive (β = 0.077, *p* < 0.05). As shown in Figure 4, the negative effect of data vulnerability on psychological comfort was mitigated when industry self-regulation was perceived to be at a high level. Hence, H6 was supported. The interaction term between government legislation and data vulnerability was again significant and positive (β = 0.078, *p* < 0.01). As shown in Figure 5, when the amount of government legislation was perceived to be high, the negative effect of data vulnerability on psychological comfort was significantly decreased. Thus, H7 was supported. A summary of the hypothesis testing results is presented in Table 5.

**TABLE 5:** Summary of hypotheses testing.

Hypotheses	Results
**H1:** Data vulnerability → Psychological comfort	Supported
**H2:** Psychological comfort → Continuance intention	Supported
**H3:** Data vulnerability → Psychological comfort→ Continuance intention	Supported
**H4:** Data vulnerability × Privacy policy→ Psychological comfort	Supported
**H5:** Data vulnerability × Privacy protection technology → Psychological comfort	Supported
**H6:** Data vulnerability × Industry self-regulation → Psychological comfort	Supported
**H7:** Data vulnerability × Government legislation → Psychological comfort	Supported

## Discussion and Conclusion

### Discussion

This study examined the influence of OHC users’ data vulnerability on their psychological comfort and continuance intention, as well as the mediating effect of psychological comfort on the relationship between data vulnerability and continuance intention, and the moderating effect of four types of institutional assurance approaches, namely privacy policy, privacy protection technology, industry self-regulation, and government legislation, on the relationship between data vulnerability and psychological comfort.

First, our results showed that data vulnerability could significantly reduce psychological comfort, which suggests that data privacy is a critical negative factor affecting OHC users’ psychology. Second, the results of our mediation analysis showed that psychological comfort negatively mediated the effect of data vulnerability on the participants’ continuance intention. This result is similar to those in previous research (Martin et al., 2017; Liyanaarachchi et al., 2021) and confirms that data vulnerability has negative effects on individuals’ psychology and behavior. It also suggests that psychological comfort plays an important mediating role in the mechanism by which data vulnerability affects continuance intention. Third, this study found that privacy policy, privacy protection technology, industry self-regulation, and government legislation positively moderated the effect of data vulnerability on psychological comfort. This result suggests that institutional assurance approaches play important roles in improving OHC users’ psychological comfort, as they can effectively weaken the negative effect of data vulnerability on users’ psychology. It also suggests that the success of OHCs relies on institutional assurance approaches provided by different OHC stakeholders.

### Theoretical Contributions

This study makes several theoretical contributions to the literature. First, our research extends the data privacy literature by introducing data vulnerability in the OHC context to elucidate individuals’ behavioral intentions, and by finding that OHC users’ data vulnerability can negatively affect their psychological comfort and continuance intention. Although studies have devoted great effort to exploring privacy issues in the online health environment, most have focused on the antecedents and outcomes of users’ privacy concerns (Zhang et al., 2018; Xu, 2019). In addition, while research on vulnerability has examined the influence of consumer vulnerability, legal vulnerability, financial vulnerability, and technology vulnerability (Baker et al., 2005; Kim, 2019; O’Connor et al., 2019; Lee, 2020), few studies have focused on the effect of data vulnerability. By integrating the vulnerability and data privacy literature, this work extends the application of the data vulnerability concept to the OHC context and offers a unique angle from which to understand and analyze data privacy issues in the online health environment.

Second, this study applied psychological comfort theory to explain how OHC users’ data vulnerability influences their continuance intention. While studies have suggested that psychological comfort plays a significant role in affecting the behavior of individuals (Akhtar et al., 2019), most have focused on the contexts of mobile applications, online tourism services, and e-commerce (Sutanto et al., 2013; Liang et al., 2018; Robinson, 2018; Yang et al., 2020). In contrast, this study examined users’ psychological comfort in disclosing their personal information in the context of OHCs and found that it negatively mediated the influence of data vulnerability on continuance intention. We therefore enrich the psychological comfort literature and provide a new perspective from which to understand the impact of data vulnerability.

Third, this research examined whether the four institutional assurance approaches of privacy policy, privacy protection technology, industry self-regulation, and government legislation could reduce the negative effect of data vulnerability on psychological comfort. Our results showed that they all positively moderated the relationship between data vulnerability and psychological comfort. Although studies have identified different institutional assurance approaches (Xu et al., 2011; Gong et al., 2019; Yang et al., 2020), no study has considered the influence of privacy policy, privacy protection technology, industry self-regulation, and government legislation simultaneously. Furthermore, most studies have only examined the direct effects of these approaches (Gong et al., 2019; Wang et al., 2019) and overlooked their potential moderating effects. By exploring the mitigating effects of privacy policy, privacy protection technology, industry self-regulation, and government legislation on the relationship between data vulnerability and psychological comfort, this study not only adds to the understanding of institutional assurance mechanisms, but also offers a unique perspective from which to learn how to reduce the negative impact of data vulnerability.

### Managerial Contributions

This research also has managerial implications for different OHC stakeholders. First, this study found that privacy policy and privacy protection technology developed by OHC service providers were effective in reducing the negative impact of data vulnerability on psychological comfort. Consequently, we recommend that OHC service providers provide users with privacy policies that contain clear explanations of what personal data will be collected, how these personal data will be used, and what compensation users will receive if the OHC fails to maintain the safety of their personal data (Gong et al., 2019; Zeng et al., 2022). Moreover, OHC service providers should employ advanced privacy protection technology, such as blockchain technology, to protect users’ personal data and earn their trust (Abdellatif et al., 2021). OHC service providers should also inform users, in straightforward and simple language, of the technology used and how it works (Wang et al., 2019).

Second, this research found that industry self-regulation could decrease the negative impact of data vulnerability on psychological comfort. Thus, industry self-regulators are advised to formulate and publish data privacy regulations to protect OHC users against unauthorized data practices (Hemphill and Banerjee, 2021). In this scenario, OHC users are likely to have faith in the OHC industry and transfer their trust to OHC service providers (Xu et al., 2012).

Third, the results of this research also suggest that government legislation can reduce the negative effect of data vulnerability on psychological comfort. Therefore, governments should be aware of the critical role of data vulnerability in deterring individuals from using OHC services (Gong et al., 2019). To alleviate OHC users’ concerns, governments are advised to establish suitable data privacy legal frameworks to regulate OHC service providers’ behavior and provide safe macroenvironments for OHC use (Hong et al., 2021).

### Limitations and Future Research

Despite its contributions, this study also has some limitations. First, this research used subjective survey data to test the conceptual model and hypotheses. Future research could collect secondary data on OHCs (e.g., Wei and Hsu, 2022) or adopt more objective research designs to improve the validity of this study and check the research findings. Second, we only focused on a single mediating variable, that is, psychological comfort. Future research could explore other mechanisms by which data vulnerability affects continuance intention in the context of OHCs. Third, this research only examined how to reduce data vulnerability from an institutional assurance perspective. Future studies could consider other possible mitigating factors in the context of OHCs from another perspective. In addition, future research could explore the effects of data vulnerability in other research contexts, especially in relation to data-driven machine learning algorithms (e.g., Kliestik et al., 2022a,b) and cognitive decision-making algorithms (e.g., Andronie et al., 2021; Pelau et al., 2021), which involve a large amount of personal data.

## Conclusion

Considering the rapid development of OHCs, it is important for OHCs to understand how certain factors obstruct users’ behaviors and how to mitigate these negative effects. This research explored the influence of OHC users’ data vulnerability on their psychological comfort and continuance intention, and the moderating effects of institutional assurance approaches. First, the results indicated that data vulnerability decreased psychological comfort, while the latter increased continuance intention. Second, the mediation analysis results showed that data vulnerability had a negative indirect effect on continuance intention through psychological comfort. Third, the moderation analysis results demonstrated that the institutional assurance approaches of privacy policy, privacy protection technology, industry self-regulation, and government legislation effectively reduced the negative impact of data vulnerability on psychological comfort.

## Data Availability Statement

The raw data supporting the conclusions of this article will be made available by the authors, without undue reservation.

## Author Contributions

WG designed the study. HW and NJ collected and analyzed the data. WG, HW, and NJ drafted the manuscript. All authors contributed to the article and approved the submitted version.

## Conflict of Interest

The authors declare that the research was conducted in the absence of any commercial or financial relationships that could be construed as a potential conflict of interest.

## Publisher’s Note

All claims expressed in this article are solely those of the authors and do not necessarily represent those of their affiliated organizations, or those of the publisher, the editors and the reviewers. Any product that may be evaluated in this article, or claim that may be made by its manufacturer, is not guaranteed or endorsed by the publisher.

## Footnotes

1
https://www.dxy.cn/bbs/newweb/pc/home

2
https://www.haodf.com/

3
https://www.xywy.com/
