# Supplementary material for: The Impact of Data Vulnerability in Online Health Communities: An Institutional Assurance Perspective
Source: Front Psychol. 2022 Jun 29;13:908309. doi: 10.3389/fpsyg.2022.908309 (PMC9277555; doi:10.3389/fpsyg.2022.908309)
Supplement: Supplementary file 1 [file Table_1.docx]

**Appendix Measurement items**

| **Data Vulnerability (DVU)** |
| --- |
| DVU1. The personal information that the online health community has about me makes me feel insecure. |
| DVU2. The personal information that the online health community has about me makes me feel exposed. |
| DVU3. The personal information that the online health community has about me makes me feel threatened. |
| DVU4. The personal information that the online health community has about me makes me feel vulnerable. |
| DVU5. The personal information that the online health community has about me makes me feel susceptible. |
| **Psychological Comfort (PCO)** |
| PCO1. I am comfortable providing information to this online health community in return for personalized services. |
| PCO2. I feel at ease in using this online health community to obtain personalized services. |
| PCO3. I feel reliable providing information to this online health community in return for personalized services. |
| **Privacy Policy (PPO)** |
| PPO1. I feel confident that this online health community’s privacy statements reflect its commitments to protect my personal information. |
| PPO2. With its privacy statements, I believe that my personal information will be kept private and confidential by this online health community. |
| PPO3. I believe that this online health community’s privacy statements are an effective way to demonstrate its commitments to privacy. |
| **Privacy Protection Technology (PPT)** |
| PPT1. I believe this online health community is equipped with privacy protection technology. |
| PPT2. I believe this online health community has reliable privacy protection technology. |
| PPT3. I believe this online health community is secure in privacy protection technology. |
| **Industry Self-Regulation (ISR)** |
| ISR1. I believe that privacy seal of approval programs will impose sanctions for this online health community’s noncompliance with its privacy policy. |
| ISR2. Privacy seal of approval programs will stand by me if my personal information is misused during and after using this online health community. |
| ISR3. I am confident that privacy seal of approval programs is able to address violation of the information I provided to this online health community. |
| **Government Legislation (GLE)** |
| GLE1. I believe that the law can protect me from the misuse of my personal data by this online health community. |
| GLE2. I believe that the law can govern and interpret the practice of how this online health community collect, use and protect my private information. |
| GLE3. I believe that the law can be able to address violation of the information I provided to this online health community. |
| **Continuance Intention (CIN)** |
| CIN1. I intend to continue using this online health community. |
| CIN2. I plan to keep using this online health community. |
| CIN3. I expect to continue using this online health community. |
| CIN4. If I could, I would like to continue my use of this online health community. |
